# Optical Microspherical Resonators for Biomedical Sensing

**DOI:** 10.3390/s110100785

**Published:** 2011-01-12

**Authors:** Silvia Soria, Simone Berneschi, Massimo Brenci, Franco Cosi, Gualtiero Nunzi Conti, Stefano Pelli, Giancarlo C. Righini

**Affiliations:** 1 MDF Lab, Istituto di Fisica Applicata Nello Carrara (IFAC CNR), Via Madonna del Piano 10, 50019 Sesto Fiorentino, Firenze, Italy; E-Mails: s.berneschi@ifac.cnr.it (S.B.); m.brenci@ifac.cnr.it (M.B.); f.cosi@ifac.cnr.it (F.C.); g.nunziconti@ifac.cnr.it (G.N.C.); s.pelli@ifac.cnr.it (S.P.); g.c.righini@ifac.cnr.it (G.C.R.); 2 Centro Studi e Ricerche e Museo Storico della Fisica Enrico Fermi, Piazza del Viminale 1, 00184 Roma, Italy

**Keywords:** microspheres, whispering gallery modes, biomedical sensors, optical resonators

## Abstract

Optical resonators play an ubiquitous role in modern optics. A particular class of optical resonators is constituted by spherical dielectric structures, where optical rays are total internal reflected. Due to minimal reflection losses and to potentially very low material absorption, these guided modes, known as whispering gallery modes, can confer the resonator an exceptionally high quality factor Q, leading to high energy density, narrow resonant-wavelength lines and a lengthy cavity ringdown. These attractive characteristics make these miniaturized optical resonators especially suited as laser cavities and resonant filters, but also as very sensitive sensors. First, a brief analysis is presented of the characteristics of microspherical resonators, of their fabrication methods, and of the light coupling techniques. Then, we attempt to overview some of the recent advances in the development of microspherical biosensors, underlining a number of important applications in the biomedical field.

## 1. Introduction

The amount of publications appeared in the last decade demonstrate the expanding utility of optical biosensors in traditional fields of life science research, drug discovery and medical applications. In particular, biomedical sensors have a vital importance in modern life. The increasing demand for rapid and reliable detection of certain biomolecules has resulted in a large variety of biosensors [1–3] and in particular of optical biosensors [4–8].

Indeed, optical measurements techniques can provide high sensitivity, compactness, fast response and real-time measurements. A great number of techniques can be used, ranging from the change of phase (refractometry) to the change of frequency (fluorescence, Raman and nonlinear optics). The phase and amplitude (absorption) changes are a direct monitoring technique whereas the frequency changes often include an indicator or marker (labeled system). Raman based detection is unique since target molecules are not labeled but the system directly detects Raman emitted light. The disadvantages of the labeled system—namely, cost and possibly reduced reactivity—are normally compensated by lower limit of detection (LOD), while in the case of direct monitoring, the disadvantage lies in the ineffectiveness of detecting small molecular weight analytes and in the sensitivity to non-specific binding. LOD is usually given in: (a) refractive index units (RIU) for label-free sensors, or (b) surface coverage in pg/mm^2^ or sample concentration in molar units for both labeled and label free sensors. A rigorous definition for LOD in resonant label-free sensors that accounts for all noise sources has been recently given [9]. Among the different optical sensor principles, none is generally superior but the preference rather depends on the application.

Evanescent wave based sensors, however, are one of the most outstanding optical sensor platforms. These devices exploit the sensing light near their surface with a penetration depth that ranges from tens to a few hundreds of nanometers; they are therefore capable of detecting changes induced by the binding of analytes within this length. In this class of sensors, microcavities sustaining Whispering Gallery Modes (WGM) are becoming very popular, as recent reviews demonstrate [10–15]. WGM resonators have different geometries with unique spectral properties, including narrow linewidth, high stability, and tunability. High quality factor *Q* and long recirculation of light in compact WGM devices are the most important features for sensing applications, where the change in *Q* or resonant wavelength can be used for measuring the change of parameters in the surrounding environment or binding phenomena on the WGM resonator surface.

It has to be noted, however, that not all the published papers referring to optical sensing in biomedical field are actually describing real biosensors. The International Union of Pure and Applied Chemistry (IUPAC) defined a biosensor as “a self-contained integrated device, which is capable of providing specific quantitative or semi-quantitative analytical information using a ***biological recognition*** element (biochemical receptor) which is retained in direct spatial contact with a transduction element”. Unfortunately, many authors still use incorrectly the word biosensor [16–19] since their devices, even if definitely interesting for potential use in biosensing applications, do not fulfill the requirement of specificity and selectivity for a given molecule.

This review is focused on biomedical sensing at the microscopic scale using spherical WGM resonators. Referring to a medical context, there is a particular strong demand for devices that can provide real time point-of-care diagnosis for various types of cancer, a wide range of pathogenic diseases [20], HIV [21] and stroke [22,23]. For example, viral detection (e.g., influenza, HIV, *etc.*) necessitates of sensor transducers with very low limits of detection [24], low false positives, and ideally it should also track the high rates of mutagenic drift. Other health threats as food-borne pathogens also require high sensitivity and specificity but are quite challenging due to larger dimensions of bacteria and more complex requirements in their detection [20,25].

## 2. General Principles: Recognition Elements, Reactive Mechanism and Sample Delivery

**Figure 1.**
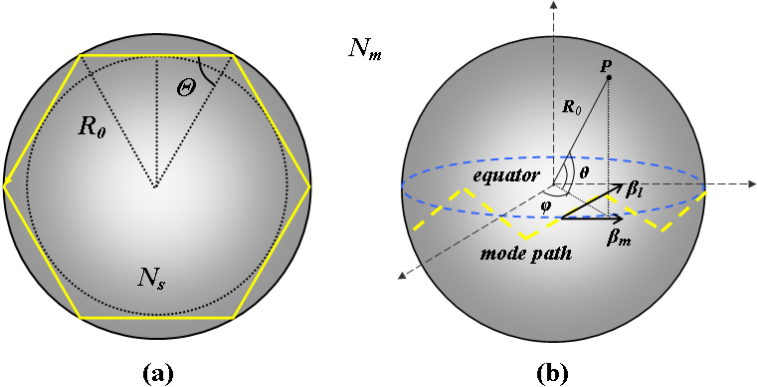
**(a)** Total internal reflection for the light rays in correspondence of the surface of the microsphere; **(b)** Spherical coordinate system and mode propagation along the equatorial plane of the sphere.

Historically, WGMs were first observed in the gallery of the cupola of St Paul’s Cathedral in London: a whisper spoken close to the wall could be heard all the way along the gallery, some 42 m to the other side. Lord Rayleigh was the first to identify the refocusing effect of the curved surface as the sound travels along the gallery; he also suggested that such modes of the electromagnetic field could find some applications due to the extreme confinement of the field [26]. With reference to the optical domain, WGMs in dielectric microspherical structures are resonant electromagnetic modes having very small mode volumes and high *Q* values. The use of the geometrical optics could be useful in order to keep a more intuitive comprehension of the mode propagation in a sphere [27]. In fact, as shown in Figure 1, a whispering gallery mode can be represented by an optical ray, trapped near the surface of the microsphere and tracing a zig-zag path around the equatorial plane (the main plane of propagation) due to the repeated total internal reflection (TIR) which occurs when *Θ > Θ_c_* = *arcsin(N_m_/N_s_)*, where *N_s_* is the refractive index of the microsphere with radius *R_0_*, and *N_m_* is the refractive index of the surrounding medium.

In one round trip, for a high number of reflections (*R_0_* >> *λ*), resonances occur when the optical path ∼2 π *R_0_* equals an integer number of wavelengths *l λ*, where *λ* is the wavelength of the light inside the microsphere medium (*λ* = *λ_0_/N_s_*, with *λ_0_* the wavelength of the light in the vacuum) and *l* is an integer number linked to the angular momentum of a circulating photon in a spherical microresonator [14]. In a spherical coordinate system as reported in Figure 1(b), *l* represents the azimuthal mode number because it is related to the electromagnetic field in azimuthal (equatorial) direction.

Moreover, other two sets of mode numbers, generally labelled with *m* and *n*, are needed in order to identify a WGM. Here *m*, which can assume the values comprised between −*l* and *+l*, represents the polar (or angular) mode number. It is referred to the amplitude of the zig-zag path and hence to the polar extent of the electromagnetic field. The number of the maxima for the field polar component, which is perpendicular to the equatorial plane and comprised between the two poles, is equal to *l* − *m* + 1. The so-called “fundamental mode”, characterized by the presence of only a maximum in the polar direction, is given by *m* = *l*. Due to the way the *θ* angle has been defined [see Figure 1(b)], this means that the fundamental mode occurs when the inclination to the equatorial plane is the smallest (*θ* ≅ 0). Otherwise, when *m* = 0, the inclination is orthogonal with respect to that of the equator. Hence, with a decrease of the polar (angular) mode number *m*, the WGMs propagate on a more tilted circular plane. The radial mode number *n* takes into account the number of field maxima in the radial direction. When *l* = *l*_max_ = 2 *m* ≈ *k_0_ N_s_ R_0_* (with *k*_0_ = 2π/*λ*_0_ the wave number in the vacuum) there is only a maximum in the radial direction (*n* = 1) and, generally, the radial field peak is close to the surface of the spherical microresonator. Modes with decreasing values of the azimuthal mode number *l* present an ever higher number of field maxima in the radial direction (*n* > 1).

The resonant wavelength of a microsphere is fixed by *l* and *n*. For instance, WGMs with same values for the mode numbers *l* and *n* and an arbitrary *m* are characterized by having the same resonant wavelengths and propagate along different circular planes with different inclinations [28]. The component of the propagation constant parallel to the surface of the sphere, in the direction of the zig-zag path, is given by $\beta_{l}=\sqrt{l(l+1)}/R_{0}$ and its projection onto the equator along the propagation direction is described by *β_m_* = *m/R*_0_.

**Figure 2.**
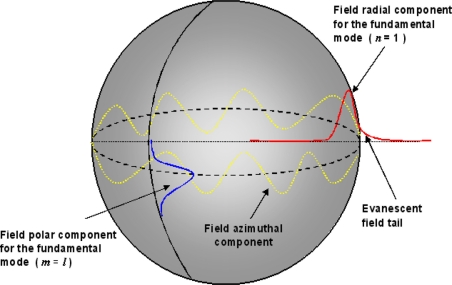
Spherical mode fields for the fundamental (n = 1) WGM.

Under the assumption that the direction of polarization associated with the electromagnetic field can be considered constant through all points in space of a spherical coordinates system, the Helmholtz equation is separable and the corresponding field solution can be expressed in the following form:

(1) $$\Psi_{l,m,n}(\gamma ,\theta ,\varphi ,)={N\psi }_{\gamma }(\gamma )\psi_{\theta }(\theta )\psi_{\varphi }(\varphi )$$

where *N* is the normalization constant and *ψ_r_*, *ψ_θ_* and *ψ_φ_* are the radial, polar and azimuthal contributions of the field as expressed in [28]. Briefly, the radial component *ψ_r_* of the total electromagnetic field is described by a spherical Bessel function inside the microsphere (r ≤ *R_0_*), while externally (r > *R_0_*) the field decays exponentially away from the microresonator surface. The polar contribution *ψ_θ_* is often expressed by spherical harmonics associated to Legendre polynomials while the azimuthal dependence *ψ_φ_* is given by a linear combination of periodical functions *sin*(*mφ)* and *cos*(*mφ)*. Figure 2 shows a qualitative distribution of the radial, polar and azimuthal field components in the case of the fundamental WGM. The presence of an evanescent field tail at the microsphere boundary explains the possibility to excite the WGMs inside the spherical microresonator by means of suitable coupling systems.

In biosensing applications the spherical microresonator is usually immersed in an aqueous solution and its surface is coated by a thin layer, needed to anchor the analyte. The effect of a high-refractive index layer is to radially compress the WGMs and to increase the evanescent field inducing an enhancement of the device sensitivity [29].

The efficient coupling of light in or out of a microsphere requires the use of near field coupling: the evanescent field of a phase-matched optical waveguide should overlap with the evanescent field of a WGM. Selective excitation of high-*Q* WGMs is possible through the use of an adjacent waveguide [30,31], a prism under total internal reflection [32,33], a tapered fiber [34–36] or an angle-polished fiber [37]. An overview and analysis of the pros and cons of these different coupling systems is given in our previous paper [14].

**Figure 3.**
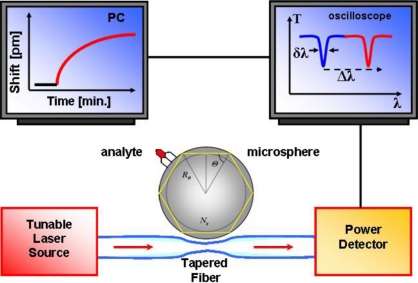
WGM resonator detection system (bottom); resonance shift after analyte binding to the surface of the microsphere and sensorgram showing the resonance signal change with time (top-left).

If scattering losses at the boundary of the microsphere are minimal and absorption of light in the transparent material is very low, then the photons are able to circulate on their orbit several thousand times before exiting the microsphere by loss mechanism. This long lifetime of the confined photons is associated to a long optical path length because of the resonant nature of the phenomenon. When a micro- or nano-metric object like a bacterium or a molecule is brought in contact with the confined circulating light, the interaction will be resonantly reinforced (reactive mechanism). A frequency shift of the resonances occurs when the radius and/or the refractive index of the sphere change; as the analyte aggregates at the surface, it interacts with the evanescent part of the WGM field inducing a change in the *Q* factor or a shift in the wavelength (Figure 3). These shifts reflect minute refractive index changes on the WGM resonator (WGMR) surface, typically down to 3 × 10^−7^ RIU in bulk [38].

Using geometrical optics, the resonant wavelength changes can be written as: *Δλ/λ* = *ΔR_0_/R_0_* + *ΔN_s_/N_s_*. Such an heuristic approach is not valid when considering random coverage by complex molecules like proteins [39,40]. Biomolecules binding to the WGMR surface shift the resonance due to their excess of polarizability, α*_ex_*, that is proportional to the molecular weight:

(2) $$\frac{\Delta \lambda }{\lambda }=\alpha_{\mathrm{ex}}\;\;\;\frac{\sigma }{\varepsilon_{0}\left(N_{s}{}^{2}-N_{m}{}^{2}\right)R_{0}}$$

where *σ* is the surface density of molecules forming a layer, and *N_s_* and *N_m_* are the refractive index of the sphere and the medium, respectively. Equation (2) describes only the shift but not the LOD. More detailed discussions about first order perturbation theory for obtaining the shift can be found in [41,42]. One can assume that the field of a particular biomolecule is not influenced by its neighbors [43] and take into account that the lowest surface density that can be detected depends on the resonance line width δλ (or a fraction F of the linewidth), which in turn depends on the quality factor *Q* = λ/δλ [11]. Accordingly, the LOD of surface density may be given by [11,21]:

(3) $$\sigma_{\mathrm{LOD}}=\varepsilon_{0}R_{0}\;\frac{\left(N_{s}{}^{2}-N_{m}{}^{2}\right)F}{\alpha_{\mathrm{ex}}Q}$$

The requirements for a biosensor are selectivity, sensitivity, stability and reversibility. These are mostly provided by the biochemical receptor (namely, antibody, antigen, DNA, enzyme, or aptamer), the sensitivity being also provided by the quality of the optical platform. Of the same importance are high signal-to-noise-ratio (SNR), short response time, low LOD, integration capabilities and high sensitivity at low cost in real samples. For example, a common noise source in a WGM resonator is the temperature fluctuation, which results in thermo-optic and thermo-mechanic effects. Even though some authors claimed that the thermo-optic effect can greatly enhance the LOD, allowing one to go down to single molecules, it has been proven that thermo-optic effects are far too small for enhancing the LOD [44]. In the same paper [44] a theory for comparing reactive and thermo-optical mechanism is given. The thermo-optical mechanism is due to the heating of the protein layer through linear absorption and its transfer to the WGM resonator. This local heating is proportional to the imaginary part of the polarizability and causes an additional shift of the resonant frequency. The authors concluded that such a thermal effect is several orders of magnitude smaller than the reactive mechanism and therefore cannot be used as enhancement mechanism in label-free biosensing. A previous paper from the same group [45] showed how a photonics mechanism, the WGM Carousel, enhances nanoparticle detection in biosensing. The Carousel is an optical trap that considerably enhances the rate of transport to the sensing region, by controlling the ionic strength of the buffer solution; the optical trapping power particles are shown to bind preferentially within the Carousel. A self-referenced WGM biosensor has been employed in order to reduce noise (thermal, non-specific binding, *etc.*) [46]. Table 1 summarizes the performances and the lowest LOD achieved by microspherical WGMR based biosensors.

Of critical importance for producing effective biosensors is the surface functionalization or chemical modification of the transducer’s surface. Proteins, for instance, adhere to any glass surface, thus increasing the unwanted non-specific effects. This crucial layer, however, has to be very thin, in the range 10–100 nm (below the evanescent penetration depth) and homogeneous in order to preserve the high quality of the transducer. There are several ways of functionalizing the surface of a biosensor; the choice of the technique should take into account several parameters: high specificity, high affinity, stability and be detectable by an optical signal. Among the various approaches, the most common ones are based on the silanization of the glass surface through covalent binding of the silane groups with the glass surface and on the use of biotin and/or streptavidin layers. The former approach shows a reduced non-specificity and enables a further functionalization with ligands or receptors [22,47–49], whereas the latter one is based on a high affinity binding with streptavidin and biotinylated molecules [50–52]. Very recently, we demonstrated the feasibility of using polymer thin layers as functionalizing agents [53].

**Figure 4.**
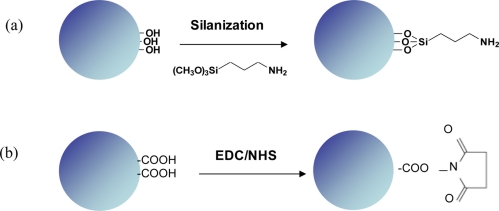
Functionalization of a WGM sensor: **(a)** a silane agent is used. In this case, the microsphere surface is previously functionalized with primary amine groups. In a second step, the receptors can be covalently bound to these groups; **(b)** after functionalization with Eudragit® the carboxyl groups (COOH) are activated with EDC/NHS chemistry.

After silane and/or polymeric functionalization, the surface can be further modified for attachment to the recognition element of interest. This bioconjugation protocol is usually based on ester chemistry. 1-Ethyl-3-[3-dimethylaminopropyl] carbodiimide hydrochloride (EDC) and *N*-hydroxysuccinimide (NHS) are commonly used to activate the functionalized surface [21,53]. NHS reacts with primary amines on a recognition element to form a stable amide bond. Figure 4 shows an example of silanization [Figure 4(a)] process and the EDC/NHS bioconjugation protocol [Figure 4(b)].

**Figure 5.**
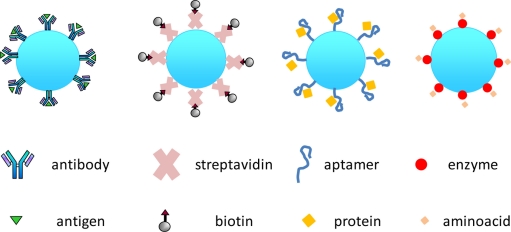
Top: Schematic representation of a WGM biosensor, resulting from the union of a WGM resonator and a sensing layer. Middle row: main ligands or receptors (antibodies, streptavidin, aptamers, enzymes). Bottom: main analytes (antigens, biotin(ylated) proteins, aminoacids).

Figure 5 shows a sketch of a biosensor based on a functionalized WGM resonator, interfaced with the biological recognition element. The middle row in Figure 5 shows a schematic representation of the most commonly used biological recognition elements: antibody, streptavidin, aptamer and enzyme; the bottom row sketches the corresponding analytes: antigen, biotin/biotinylated proteins, proteins, and amino acids. Antibody-based sensors are also called immunosensors while aptamer-based sensors are called aptasensors. The antibody shows high affinity and specificity towards the antigen, due to their molecular complementarities. Enzymes are specific in both the reaction they catalyze and the substrate they recognize and are subject to regulation of their activity by other molecules. Therefore, the use of enzymes can be advantageous owing to enzyme specificity and to the amplification phenomena given by enzyme catalysis. A major disadvantage is the dependence of the enzyme activity on physical and chemical environments. Aptamers are functional molecules selected *in vitro*. Aptamers have high specificity and affinity, and even small changes in the target analyte may disrupt the binding; they can in principle be selected *in vitro* for any given target, ranging from small molecules to large proteins and even cells, thus making it possible to develop a wide range of aptasensors.

Biosensors can also be classified depending on their assay format. The main formats are direct and indirect, which typically correspond to a label-free [50] and labeled [51] biosensor, respectively. Competitive assays are a third format commonly used in labeled systems where the target analyte has small molecular weight. In this format a labeled antigen competes with a non-labeled one for the available binding sites. The transducer can differentiate the relative amount of antibody binding sites that are occupied by the analyte. This results in a sensor signal that is inversely proportional to the analyte concentration. Competitive assays are very complex and long since they are multi-stepped. No competitive assay has ever been published with WGMR as transducers.

## 3. Experimental Set-Up

**Figure 6.**
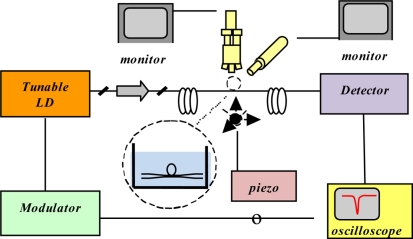
A schematic diagram of the experimental arrangement employing a bi-conical tapered fiber coupler.

As already said, resonant wavelengths can be selectively excited and detected by using a narrow linewidth laser and scanning over a range of wavelengths. Such a laser can be finely swept at very low frequency around a resonance by a few GHz. The light transmitted through the coupler-WGMR system (e.g., using a tapered fiber coupler) is monitored at the output of the taper by a detector connected to an oscilloscope, as shown in Figure 6.

**Figure 7.**
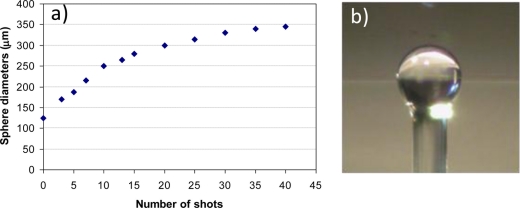
**(a)** Size of the microspheres produced at the tip of a standard 125 μm telecom fiber, as a function of the arc shots in a commercial fiber fusion splicer. **(b)** Optical image of a microsphere with a diameter of about 250 μm. In the background (out of focus) one can see the coupling bi-conical tapered fiber.

The microspheres can easily be fabricated directly on the tip of a standard telecom fiber by using a fiber fusion splicer, CO_2_ laser fusion or gas flame. In the first approach, a cleaved tip of the fiber is inserted in one arm of the splicer and a series of arcs are then produced. The tip partially melts and surface tension forces induce the spherical shape. The size of the sphere increases with the number of arc shots, till it tends to saturate at a diameter of about 350 μm (Figure 7). In order to obtain spheres with diameters below 125 μm, which is the size of the clad of a standard fiber, the fiber is first tapered (e.g., by heating and stretching the fiber itself till it breaks). The minimum diameter we can obtain using this method is about 40 μm [54].

Arnold’s group fabricated microspheres by rotating a fiber in an oxygen-propane micro-flame; the obtained spheroids have equatorial radii between 75 and 200 μm and result to be oblate with an eccentricity ∼6% [21]. Eccentricity increases the number of excited modes [55] but the perturbation and variational methods [56] still apply as long as the perturbation does not change the eccentricity.

**Figure 8.**
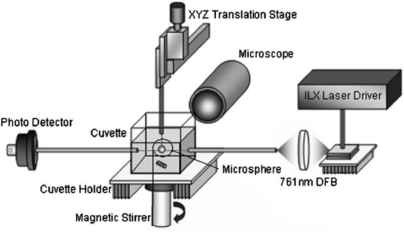
Experimental set-up based on a disposable cuvette of 1 cm^3^ volume, temperature controlled and magnetically stirred. Reprinted with permission from [57] © 2005, American Institute of Physics.

**Figure 9.**
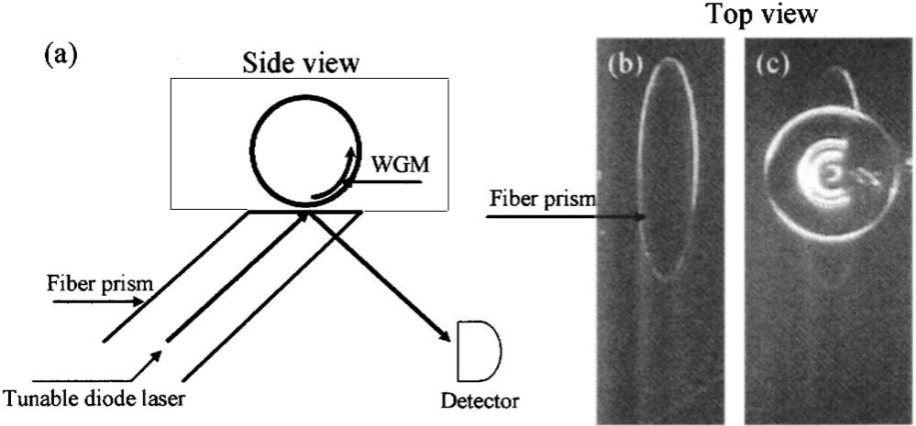
**(a)** Schematic of the microsphere and fluidic cell (side view); **(b)** top view of the fiber prism and of **(c)** the fused silica microsphere placed in contact with the fiber prism. Reprinted with permission from [38] © 2005, American Institute of Physics.

Sample delivery is also a critical step in a biosensor device. Biochemical samples are generally in the form of aqueous solution; microfluidics is one of the most commonly used methods of sample delivery. Ideally, fluidics design should take into account sample injection and drainage, reduction of sample volume and detection time. Integration of microsphere(s) and coupling system is a quite challenging task; several attempts, however, to integrate them with fluidics have been made. In early works [48,57] small cuvettes and fluidic cells were used. The microsphere and the coupling taper were immersed in 1 cm^3^ of buffer solution; the fluidic system was temperature controlled and magnetically stirred for sample mixing (see Figure 8). Small fluidic cells have been fabricated by choosing as a coupling system either an angle-polished fiber prism [38] or a bulk prism [52,58] and then using as a bottom window of the cell the prism itself. Figure 9 shows the angle-polished fiber prism approach.

**Figure 10.**
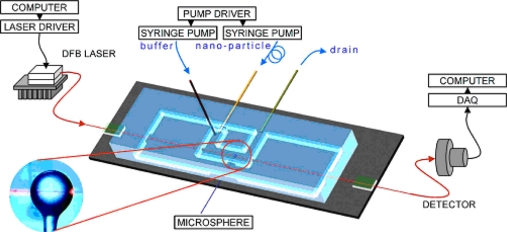
Integrated WGMR and microfluidic system for biosensing. Reprinted with permission from [59] © 2007, American Institute of Physics.

When measuring adsorption and desorption rates, smaller motorized microfluidic cells are more convenient. A microfluidic flow system that incorporates the microsphere and the taper system is shown in Figure 10 [21,59]. In this system, the taper is bound to a glass slide by UV adhesive and acts as a foundation. This foundation is placed on top of a thermoelectric stage for temperature control. The microfluidic cell is made by replica molding of a master in polydimethylsiloxane (PDMS). The cross section of the microfluidic channel is 1.5 mm × 2.5 mm and has a total volume of 100 μL [21]. Two computer driven syringe pumps flow the sample and the buffer for washing in between two sample injections, in order to avoid cross-sample contamination.

## 4. Biomedical Applications

### 4.1. Sensors Based on Passive Microspheres

The first experiment that demonstrated the quantitative use of WGM spherical biosensor dates back to 2002 [50]. In this early paper, specific detection of proteins was proven. A year later, the same group demonstrated the feasibility of multiplexed DNA quantification [47] and single-nucleotide polymorphism analysis by hybridization. An unprecedented LOD of 6 pg/mm^2^ was demonstrated. Specific and multiplexed DNA detection was performed by using two microspheres, allowing to discriminate a single nucleotide mismatch. Such technique could be of great use in detection of mutation of genes involved in cancer development without target labeling.

**Figure 11.**
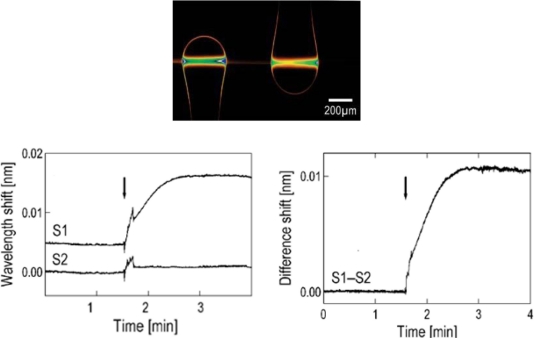
Top: Optical image of two microspheres coupled to a taper, showing two resonances orbiting inside the spheres. Bottom: Single nucleotide mismatch detection; left: time traces of the two microspheres, the perfect match sequence gives a signal 10 times larger; right: difference signal, allowing to achieve SNR = 54. Reprinted with permission from [47] © 2003, Elsevier.

Figure 11 shows an optical image of the two microspheres coupled to a taper running horizontally through the image in the background, and therefore scarcely visible. Two resonances of light orbiting inside each microsphere are shown. On the bottom of the figure, sensorgrams are shown. S1 corresponds to the microsphere modified with a biotinylated 11-mer oligonucleotide (5’-biotin-CTATCTCAGTC); the oligonucleotide immobilized in S2 differed by a single nucleotide (5’-biotin-CTATATCAGTC). The arrow in the plots indicates when the oligonucleotide complementary to the sequence of the one immobilized on S1 is injected (left plot). Spikes are due to transient temperature and refractive index fluctuations. The difference of the signals (S1–S2) allows to identify the mismatch with a signal-to-noise ratio (SNR) of 54 (right plot). The advantage of using two microspheres is obvious, as one can remove all common noise in experiments.

A similar scheme has been also reported very recently [46]. The authors have theoretically demonstrated the capabilities of the biosensor consisting of two microspheres coupled to each other in a way that they behave as “photonic molecules”. They can thus discriminate between bulk index changes and specific/nonspecific surface binding events. The authors proposed as detection method the tracking of the bonding and antibonding optical modes.

**Figure 12.**
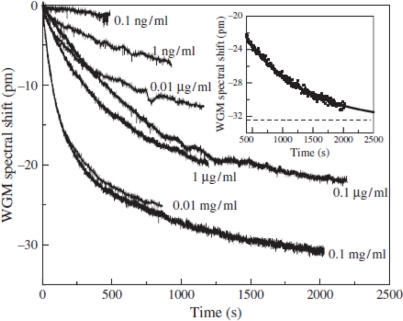
Sensorgrams for BSA cleavage at various concentrations of trypsin. Inset: exponential fit decay for the 0.1 mg/mL curve in the time range of 400–2,000 s; the decay constant is 950 s, and the dashed line indicates the baseline (−32.6 pm). Reproduced with permission from [48] ©2005 American Scientific Publishers.

An important clinical application for WGM biosensors is to detect proteolytic activity [48]. A number of neurotoxins, metabolic and cardiovascular markers participate in physiological processes through protein cleavage. In the work of Fan’s group, the BSA-trypsin pair is taken as a model system to model protein or peptide cleavage. BSA contains 60 lysine and 26 arginine residues that can be cleaved by trypsin. Partial removal of BSA molecules in presence of trypsin results in a blue shift of the resonances. The LOD was 10 pg/mL, obtained within 15 minutes. Figure 12 shows the sensorgrams for BSA cleavage at various concentrations of trypsin. As shown in the inset, an excellent exponential decay fit is obtained for times greater than 400 s, showing the maximal achievable cleavage corresponding to a shift of −32.6 pm.

**Figure 13.**
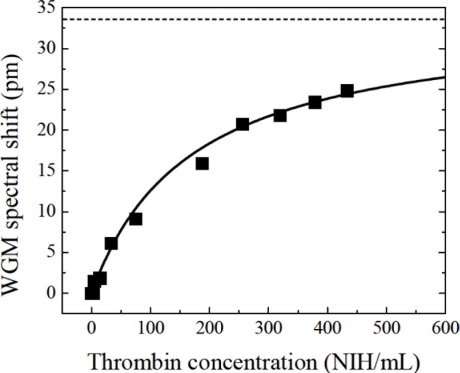
WGM spectral shift *vs.* thrombin concentration. Solid line: theoretical fit; dashed line: WGM shift for maximal thrombin binding. Reproduced with permission from [22] © 2006 MDPI.

An aptamer based microspherical biosensor has been demonstrated for thrombin detection [22]. The minimum LOD experimentally tested was 5 NIH units/mL of thrombin (1 NIH unit = 0.324 ± 0.073 μg), but a LOD of 1 NIH/mL could be estimated from the measurements performed. Control experiments to confirm the specific binding were carried out. The interaction between aptamer and thrombin can be described by:

(4) $$\delta \lambda =\frac{{\delta \lambda }_{\max}[\text{thrombin}]}{[\text{thrombin}]+K_{d}}$$

where K_d_ is the dissociation constant, [thrombin] is the concentration of thrombin and δλ_max_ is the WGM shift when the microsphere surface is saturated with aptamer molecules. Figure 13 shows the fitting of the data by Equation (4) with K_d_ = 170 NIH/ml\L (1,000 nM) and δλ_max_ = 34 pm.

**Figure 14.**
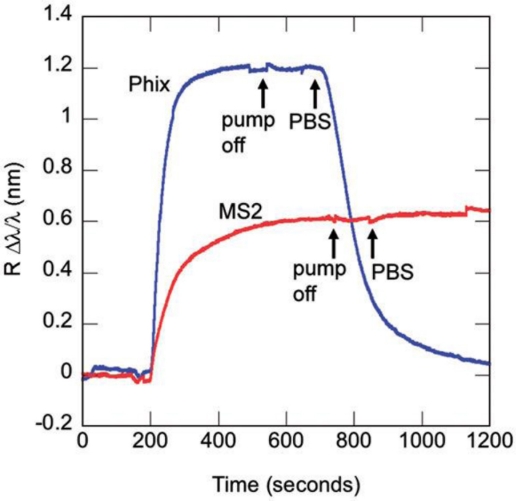
Comparison between Phix174 (blue line) and MS2 (red line) detection experiments on the same microspherical WGMR [21]—Reproduced by permission of The Royal Society of Chemistry.

Identification of whole viruses has also been demonstrated [21]. The format used is that of a direct immunosensor with the complementary antibody anchored to the surface of the microsphere. The model system here is a RNA virus known as MS2, that kills *E. coli* but is harmless to humans. The negative control is Phix174, another E. coli virus. Phix174 and MS2 are icosahedral viruses like HIV and their coat proteins contain different epitopes; Phix174 should therefore not specifically bind to the surface anchored anti-MS2. Figure 14 shows the sensorgrams for both analytes. Phix174 was injected into the fluidic cell at concentration 2.5 pM: the resonance frequency shifted toward longer wavelengths and reached equilibrium before switching off the pump. 100 s later, PBS was injected into the cell and the wavelength dropped to the baseline, indicating the lack of covalent binding to the anti-MS2; in the case of specific binding, in fact, after washing with PBS, there would not be any evidence of desorption. The arrows in the figure indicate the time when the pump was turned off and PBS was injected. The same authors pushed further the LOD in the case of influenza A, down to single virions, but that result was obtained for non-specific sensing [24].

WGMs can also be excited with a polarized optical field. Such versatility can be very useful for measuring anisotropies in the biological layers covalently bond or adsorbed to the surface of the WGM resonator. Molecules attach themselves in preferred orientations. This preferred orientation can be determined by measuring the shift ratio between the fields parallel and perpendicular to the surface, and comparing it with the theory [60]. The technique has been applied to study conformational changes in self-assembled monolayers such as biological membranes [61]. Even though the demonstration has been performed in a model system like bacteriorhodopsin, it has a very exciting future application to protein folding studies. As well known, there are many diseases that result from misfolding of proteins or anomalous aggregations, such as Parkinson’s and Creutzfeldt-Jakob’s diseases, among many others.

WGMRs, moreover, can be combined with other optical techniques, such as surface-enhanced Raman scattering (SERS), in order to measure the fingerprint of the bound molecules. It has been demonstrated theoretically that SERS can happen in WGM resonators without plasmons [62], at least in the case of microspheres of high refraction index and small radius.

**Figure 15.**
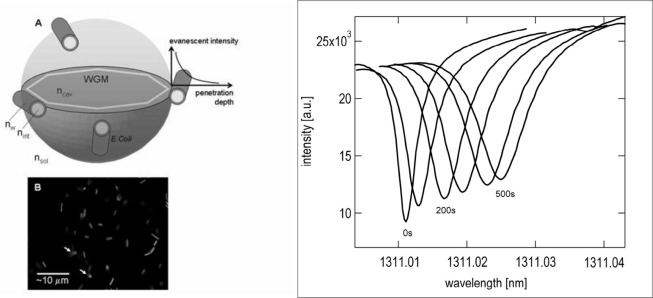
**(A)** Sketch of a WGM confined at the equator of the microsphere. N_cav_, n_sol_, n_w_, n_int_ are the refractive indexes of cavity, surrounding aqueous solution, the bacteria cell wall and interior (protoplasm) of the bacteria; **(B)** Fluorescent image of green fluorescent protein (GFP)-labeled bacteria bound to the surface. Only few bacteria (arrows) are bound at their tip; Right: Shift and broadening of the linewidth of the resonance due to adsorption of *E.coli*. Reproduced with permission from [20] © (2007), Optical Society of America.

Microspherical resonators also provide an useful tool to study larger biological materials, such as micrometer sized cells and bacteria. Analytic results were obtained for rodlike bacteria, relating the shift in wavelength and the broadening of the linewidth due to scattering [20]. These results were confirmed by measuring *E. coli* (model system) with sensitivity of about 100 bacteria/mm^2^. The shift and broadening of the linewidth can be seen in Figure 15, where a series of transmission spectra obtained for successive time intervals during absorption are shown on the right. In this case the measurement is based on non-specific adsorption. Figure 15(A) shows a WGM excited at a wavelength of about 1,300 nm and its evanescent tail. Figure 15(B) shows a fluorescent image of the genetically modified bacteria adsorbed into the surface of the microsphere.

### 4.2. Sensors Based on Active Microspheres

Active microspheres were proposed as sensor transducers with enhanced sensitivity [63]. The first demonstration was based on polymer microspheres doped with a gain medium, namely a fluorescent dye like rhodamine 6-G. The idea behind was that such microspheres could achieve narrower linewidths at lasing condition, and therefore increase by several orders of magnitude the *Q*-factor. The authors calculated that the LOD of active polystyrene microspheres could be 10^−10^ RIU, beating surface plasmon resonance sensors.

The experimental demonstration of non-specific sensing with active microspheres was published recently [18]. Nile red doped polystyrene beads were pumped by a picosecond pulsed laser and in the stimulated regime there was an 8-fold enhancement of the SNR and a 3-fold enhancement of the *Q*-factor.

**Figure 16.**
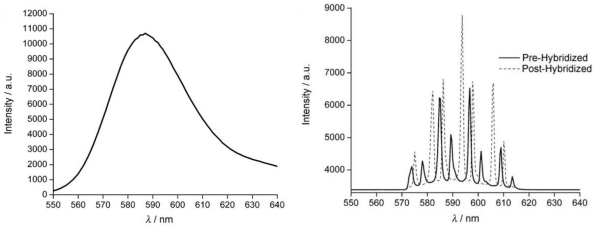
Fluorescent spectrum of the TMR-dyed microsphere. Right: WGM emission spectra of the same microsphere acquired pre- and post-complementary strand treatment. The peaks get red shifted following 90 s exposure to cDNA probe (dashed line). Reproduced with permission from [49]. ©Wiley-VCH Verlag GmbH & Co. KGaA (2007).

Specific detection of oligonucleotides was demonstrated by using silica microspheres functionalized with a fluorophore (tetramethylrhodamine—TMR) and a dense monolayer of single-strand of oligonucleotides [49]. The specific binding of the complementary strand caused shifts in the emission spectrum of the microsphere that was detected by an optical microscope and a CCD detector (Figure 16). The hybridization kinetics and the denaturation of the double strand DNA were monitored at high temperatures; it was proved that hybridization was reversible. The same authors obtained similar results with quantum dots embedded into microspheres. The WGMs were broader and less sensitive but no photobleaching occurred.

Other authors have demonstrated that quantum dot-embedded microspheres could enhance up to five times the theoretical sensitivity [64]. Two-photon excited luminescence improved the detection of WGM by localization. In bulk refractometric measurements the authors detected changes of 2.5 × 10^−4^ RIU, five times greater than the calculated sensitivity. In non-specific sensing of protein layers (bovine serum albumin and thrombin) adsorbed to the Q-dot microsphere, the enhancement was almost three times [23]; in this case, however, the authors did not use two photon excitation of the luminescence of the CdSe/ZnS Q-dots.

Very recently, a new absolute WGM-based sensing method has been proposed that does not require calibration, reference measurements or any prior knowledge about the microsphere radii [25]. Only one spectrum acquisition per fluorescent microsphere is needed. The authors proposed the use of commercially available free floating functionalized fluorescent microspheres, thus reducing significantly the sample preparation. The proposed analyte was constituted by elongated spores of *Bacillus atrophaeus subsp. Golbigii* (B. golbigii). The authors used two different parameters, *i.e.*, the apparent refractive index and the calculated mean TE-TM mode spacing, for successful detection of the spores.

Fluorescent dye-doped polystyrene microspheres have also been proposed as remote sensor for *in-vitro* sensing of biomechanical forces [65]. The stress induced by a live cell during endocytosis, corresponding to the incorporation of the microsphere into the cell, resulted in the microsphere deformation and consequently into a broadening and a blue shift of the WGM resonances. The authors demonstrated the feasibility of the method, and found an unexpectedly high stress, attributed to a so far undetermined stress component induced by the cytoskeletal machinery during particle incorporation. Further work would be needed in order to elucidate the stress mechanism. Since it has been found that the biomechanical properties of the cytoskeleton in cancer cells differ significantly from those of non-malignant cells [66], the proposed use of a microspherical WGMR as a non-destructive probe of cell stress may be of interest for early cancer diagnosis too.

## 5. Conclusions

Several laboratory experiments have demonstrated the large potential of the WGMR based sensors in the biomedical field. Microspherical sensors have been the first devices to permit demonstration of all-optical label free single molecule detection and to enable the study of non-equilibrium dynamics of molecular recognition events and conformational changes. The combination of WGMR and SERS led to the feasibility proof of spectroscopic detection of the fingerprint of the bound analyte.

Being able to detect large targets, WGM spherical resonators can be used in cell-based assays. In this case, the membrane cell can be bound to the microsphere surface and the response of the resonator is local to the membrane events.

The future applications of WGM spherical resonators can be extended to high sensitivity detection of disease markers in point of care testing and high-throughput drug screening. More basic research studies in life sciences could include WGM spherical resonators in studies of protein folding and membrane biophysics.

Wider exploitation of the unique properties of microspherical WGMRs for biosensing would however require overcoming the still existing difficulties in integration of the microsphere-coupling system with microfluidics, in order to achieve robust devices for routine diagnostics with very small sample volumes.

## Figures and Tables

**Table 1.** A summary of some label-free WGMR biosensing results; the properties of the transducer, the target analyte, the reported LOD and the reference are indicated.

**Dielectric Microsphere** 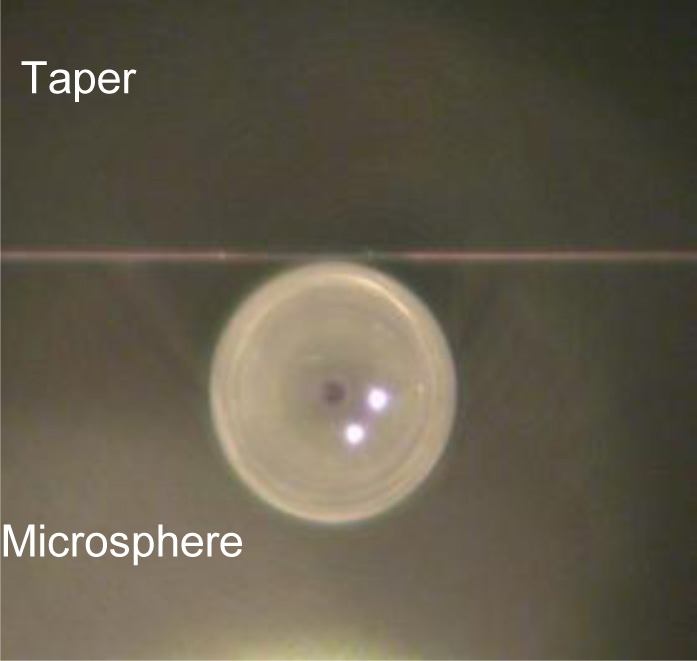	**Properties**	**Analyte**	**LOD**	**Reference**
	High Q-factor in H_2_O	Bulk solution *	10^−7^ RIU	[38]
	Multiplexed measurement	DNA	1 pg/mm^2^	[47]
	Very challenging microfluidics	Protein	10 pg/mL (trypsin) 1 unit/mL (thrombin)	[48] [22]
	Very challenging integration	Virus	1 pg/mm^2^ Single virion	[21] [24]
	Difficult mass production	Bacteria *	100 cfu/mm^2^	[20]

* non-specific binding.
